# Spatial analysis of overweight prevalence in China: exploring the association with air pollution

**DOI:** 10.1186/s12889-023-16518-6

**Published:** 2023-08-22

**Authors:** Peihan Wang, Kexin Li, Chengdong Xu, Zixuan Fan, Zhenbo Wang

**Affiliations:** 1 Key Laboratory of Regional Sustainable Development Modeling, Institute of Geographic Sciences and Natural Resources Research, Chinese Academy of Sciences, Beijing, 100101 P.R. China; 2 Key Laboratory of Land Surface Pattern and Simulation, Institute of Geographic Sciences and Natural Resources Research, Chinese Academy of Sciences, Beijing, 100101 P.R. China; 3 State Key Laboratory of Resources and Environmental Information System, Institute of Geographic Sciences and Natural Resources Research, Chinese Academy of Sciences, Beijing, 100101 P.R. China; 4 University of Chinese Academy of Sciences, Beijing, 100049 P.R. China; 5 Present Address: School of Health Policy and Management, Peking Union Medical College, Beijing, 100730 P.R. China

**Keywords:** Spatial heterogeneity, Air pollution, Overweight, Middle-aged, Elderly

## Abstract

**Background:**

Overweight is a known risk factor for various chronic diseases and poses a significant threat to middle-aged and elderly adults. Previous studies have reported a strong association between overweight and air pollution. However, the spatial relationship between the two remains unclear due to the confounding effects of spatial heterogeneity.

**Methods:**

We gathered height and weight data from the 2015 China Health and Retirement Long-term Survey (CHARLS), comprising 16,171 middle-aged and elderly individuals. We also collected regional air pollution data. We then analyzed the spatial pattern of overweight prevalence using Moran's I and Getis-Ord Gi* statistics. To quantify the explanatory power of distinct air pollutants for spatial differences in overweight prevalence across Southern and Northern China, as well as across different age groups, we utilized Geodetector's q-statistic.

**Results:**

The average prevalence of overweight among middle-aged and elderly individuals in each city was 67.27% and 57.39%, respectively. In general, the q-statistic in southern China was higher than that in northern China. In the north, the prevalence was significantly higher at 54.86% compared to the prevalence of 38.75% in the south. SO_2_ exhibited a relatively higher q-statistic in middle-aged individuals in both the north and south, while for the elderly in the south, NO_2_ was the most crucial factor (q = 0.24, *p* < 0.01). Moreover, fine particulate matter (PM_2.5_ and PM_10_) also demonstrated an important effect on overweight. Furthermore, we found that the pairwise interaction between various risk factors improved the explanatory power of the prevalence of overweight, with different effects for different age groups and regions. In northern China, the strongest interaction was found between NO_2_ and SO_2_ (q = 0.55) for middle-aged individuals and PM_2.5_ and SO_2_ (q = 0.27) for the elderly. Conversely, in southern China, middle-aged individuals demonstrated the strongest interaction between SO_2_ and PM_10_ (q = 0.60), while the elderly showed the highest interaction between NO_2_ and O_3_ (q = 0.42).

**Conclusion:**

Significant spatial heterogeneity was observed in the effects of air pollution on overweight. Specifically, air pollution in southern China was found to have a greater impact on overweight than that in northern China. And, the impact of air pollution on middle-aged individuals was more pronounced than on the elderly, with distinct pollutants demonstrating significant variation in their impact. Moreover, we found that SO_2_ had a greater impact on overweight prevalence among middle-aged individuals, while NO_2_ had a greater impact on the elderly. Additionally, we identified significant statistically interactions between O_3_ and other pollutants.

## Background

Overweight is rising rapidly, and it has become a significant threat to public health worldwide [1, 2]. In 2019, 40% of adults were overweight [3]. Numerous epidemiological studies have found that overweight is associated with various chronic diseases, including cardiovascular disease, diabetes, several cancers, etc. [4–7]. Moreover, overweight has a negative impact on the quality of life of the subjects, and it increases medical expenses, which are financially burdensome for both individuals and the government.

The prevalence of overweight in adults is rapidly increasing in developing countries and causing a significant burden [8]. China, as the largest developing country in the world, has witnessed a significant increase in the prevalence of overweight over the past 40 years, turning into the country with the largest population of individuals with overweight [9]. As a result of China’s rapid social and economic development and urbanization, the projected prevalence of overweight among Chinese adults by 2030 is 65.3%, with medical expenses associated with overweight estimated to reach approximately 61 billion U.S. dollars, which will account for approximately 20% of annual health expenditures [10]. Moreover, China is one of the world’s fastest-aging countries, with an estimated 400 million people over 65 years of age by 2050, accounting for 26.9% of the total population [11]. Therefore, overweight among middle-aged and elderly Chinese individuals is in urgent need of attention.

Several factors can contribute to overweight in middle-aged and elderly people, including diet, sleep quality, mental stress, living habits, neighborhood environment, air pollution, and socioeconomic status [12–14]. Of these factors, long-term exposure to air pollutants, such as inhalable particles (PM_10_), nitrogen dioxide (NO_2_), sulfur dioxide (SO_2_), and ozone (O_3_), has been significantly associated with high body mass index (BMI) or overweight risk [15]. Existing literature shows that these pollutants can lead to adipose tissue inflammation, oxidative stress, and metabolic dysfunction, ultimately resulting in overweight [16–18]. Another pollutant, fine inhalable particles (PM_2.5_), is known to interfere with insulin resistance and inflammation by affecting reactive oxygen species generated by NADPH oxidase, thereby affecting fat metabolism [19, 20]. Additionally, severe air pollution can reduce outdoor physical activity, which thereby reduces fat consumption and increases the risk of overweight [21]. Although many studies have investigated the influence of different pollutants on overweight, no previous studies have explored the spatial heterogeneity of such factors’ influence.

However, the existing literature demonstrates that the omission of spatial heterogeneity can lead to statistical confounding [22]. Furthermore, the comprehensive investigation of the impact of air pollution on overweight risk, considering spatial heterogeneity, remains insufficient. In this study, our hypothesis posits a positive association between air pollution and overweight, with variations expected among different pollutants. Additionally, we anticipate that these associations may be influenced by age groups and regional disparities, particularly between the southern and northern regions. Thus, the identification of the spatial effects of air pollution on overweight among middle-aged and elderly populations holds significant value in the development and implementation of regionally tailored early intervention strategies to address the issue of overweight.

## Methods

### Data of overweight

The data on overweight among middle-aged and elderly individuals in China for 2015 were obtained from the 2015 China Health and Retirement Longitudinal Study (CHARLS). This comprehensive interdisciplinary survey project was conducted by Peking University and was funded by the Natural Science Foundation of China. The primary goal of the funded project is to collect high-quality micro-data that depicts families and individuals of middle-aged and elderly individuals aged 45 and above in China. The data is then analyzed to address the issue of population aging in China, encourage interdisciplinary studies on aging issues, and support the development of relevant policies in our country based on scientific knowledge. Ethical standards were followed in obtaining informed consent from all participants. Further information about the CHARLS has been previously published by Zhao et al. [23]. Middle-aged individual was defined as those between the ages of 45 and 60, and elderly were defined as 60 years or older. In this study, the division between the northern and southern regions of China was determined based on the geographical features of the Qinling Mountains and the Huaihe River, which formed a continuous line extending from west to east.

Underweight was defined as BMI of less than 18.5; normal weight, 18.5 to 23.9; overweight, 24 to 27.9, and obesity, 28 or higher, according to the Chinese national standard [24]. The data collection for the 2015 CHARLS survey was conducted longitudinally throughout the entire year, with multiple waves of data collection. This approach was adopted to capture any potential fluctuations in individual biomarkers, including height and weight. In our study, the average level of these biomarkers was utilized as an index in the statistical unit, representing the city level. This allows us to obtain a more comprehensive understanding of the biomarker profiles of the study population. BMI was calculated using height and weight data, where BMI is defined as the quotient of height divided by the square of weight. Both height and weight measurements were obtained using a stadiometer and a scale, respectively. (The index of height and weight were collected by the standardized equipment of SecaTM213 Stadiometer and OmronTMHN-286 Scale respectively). The data on overweight prevalence among individuals, categorized by age group, were obtained by calculating the overweight prevalence rate for each individual city using raw data collected (Refer to Table 1). And our analysis will be carried out at the city level.

**Table 1:** Statistical information for overweight prevalence in different ages in north and south China

Prevalence of overweight	5%	50%	95%	mean
Middle-aged in northern China	54.55%	75.00%	85.71%	73.95%
Elderly in northern China	41.74%	66.67%	72.73%	60.27%
Middle-aged in southern China	42.67%	61.36%	77.89%	61.22%
Elderly in southern China	20.90%	48.00%	68.97%	57.39%

### Data of air pollution

Data on air pollution levels in 2015 were presented as the yearly average concentration of PM_2.5_, PM_10_, SO_2_, O_3_, and NO_2_ resulting from urban pollution. The China National Environmental Monitoring Center (CNEMC) provided this data set, which was calculated from hourly monitoring data collected by the National Environmental Monitoring Stations, as shown in Table 2.

**Table 2:** Statistical information for pollutants in the northern and southern regions of China

Region	Factor	Abbreviation	5%	50%	95%	mean
North	Fine particulate (μg/m^3^)	PM_2.5_	28.73	53.31	88.33	56.21
	Inhalable particles (μg/m^3^)	PM_10_	57.86	97.02	157.26	104.52
	Sulfur dioxide (μg/m^3^)	SO_2_	12.05	29.78	52.61	31.56
	Ozone (μg/m^3^)	O_3_	47.44	62.47	79.13	62.09
	Nitrogen dioxide (μg/m^3^)	NO_2_	19.92	34.37	47.09	33.86
South	Fine particulate (μg/m^3^)	PM_2.5_	22.63	43.09	60.99	43.50
	Inhalable particles (μg/m^3^)	PM_10_	40.00	69.50	98.10	69.77
	Sulfur dioxide (μg/m^3^)	SO_2_	8.14	17.47	34.26	18.84
	Ozone (μg/m^3^)	O_3_	40.47	56.40	74.87	57.50
	Nitrogen dioxide (μg/m^3^)	NO_2_	16.04	25.89	43.26	26.72

### Statistical analysis

The prevalence of overweight among middle-aged and elderly individuals in China was analyzed initially using both Moran’s I index and Getis-Ord Gi* statistics to identify any spatial patterns. Moran’s I index is reflective of the similarity of attribute values of adjacent regional units, also known as spatial adjacency [25]. The Moran’s I index is used in this paper to explore the spatial autocorrelation of overweight prevalence among middle-aged and elderly individuals in China. The formula is as follows:

$$I=\frac{\sum_{i=1}^{n}\sum_{j\ne 1}^{n}{w}_{ij\left({x}_{i}-\overline{x}\right)\left({x}_{j}-\overline{x}\right)}}{{S}^{2}\sum_{i=1}^{n}\sum_{j\ne 1}^{n}{w}_{ij}}$$

$${S}^{2}=\frac{1}{n}{\sum }_{i=1}^{n}{\left({x}_{i}-\overline{x}\right)}^{2}$$

$$Z=\frac{I-E\left(I\right)}{\sqrt{VAR\left(I\right)}}$$

Where: *i* = 1, …, *n*; *j* = 1, …, *n*; *i* $\ne j;{x}_{i}$ and ${x}_{j}$ are the values of the prevalence of overweight at position *i* and position *j* respectively; $\overline{x }$ is the mean of the prevalence of overweight; ${S}^{2}$ is the variance; ${w}_{ij}$ is the spatial weight matrix of the prevalence of overweight. Analysis of cold and hot spots can identify areas of high and low agglomeration values [26]. This paper utilizes this method to investigate the spatial distribution and agglomeration characteristics of overweight prevalence among middle-aged and elderly individuals in China. The formula is as follows:

$${G}_{1}*=\frac{\sum_{j=1}^{n}{w}_{ij}{x}_{j}}{\sum_{j=1}^{n}{x}_{j}}$$

Where: *i* = 1, …, *n*; *j* = 1, …, *n*; *i* $\ne j;{x}_{i}$ and ${x}_{j}$ are the values of overweight prevalence at position *i* and *j*, respectively; ${w}_{ij}$ is the spatial weight matrix of overweight prevalence.

The effect of air pollution on overweight is a long-term effect, and previous studies have indicated a stable spatial pattern of air pollution in China in recent years, with no significant changes in spatial differences over an extended period of time [27, 28]. In the study, a spatial statistic method, named Geodetector q-statistic, was employed, it treated air pollution as an ordinal category variable, allowing us to capture the relative intensity of air pollution across different regions.

Geodetector q-statistic has the ability to detect spatial variability and to reveal the underlying driving forces [22, 29], it has been widely used in field of public health [30, 31]. In the study, the individual or interaction effects of different air pollutants on overweight prevalence among middle-aged and elderly individuals in various regions were analyzed based on this method. The q-statistic, which ranges from 0 to 1, measures the impact of each air pollutant on the spatial variability of overweight prevalence. Calculate with the following formula [22]:

$$q=1-SSW/SST$$

$$SSW=\sum_{h=1}^{L}{N}_{h}{\sigma }_{h}^{2}, SST={N\sigma }^{2}$$

where: *h* = *1, 2, …, L* is the number of strata of the selected risk factors *X*; *N* and *σ*^*2*^ are the total number of samples and the variance of *Y* (prevalence of overweight of middle-aged and older Chinese adults) in the whole study area, respectively; and ${N}_{h}$ and ${\sigma }_{h}^{2}$ are the mean number of samples and the local variance of *Y* in strata *h*, respectively. Therefore, *SSW* is the within the sum of squares, and *SST* is the total sum of squares. The range of the q-statistic is 0–1; the larger the q, the stronger the effect of factor *X* on *Y*. As the q-statistics of different factors represent their explanatory power on *Y* separately, they are not additive.

Based on the method, interaction between different risk factor *Xs* can be assessed, we used it to evaluate whether factors *X1* and *X2* will increase or decrease the explanatory power of dependent variable *Y* when they act together, or whether these factors have independent effects on *Y*. The evaluation method is to first calculate the q-statistics of two factors *X1* and *X2* for *Y*: *q (X1)* and *q(X2)*, and then calculate the q-statistics of *q(X1⋂X2)* when they interact with each other, and compare *q(X1)*, *q(X2)*, and *q(X1⋂X2)*. The relationship between two factors can be divided into the following categories (Table 3).

**Table 3:** Interaction relationship between two factors

Description	Interaction
*q(X1 ∩ X2)* < Min*(q(X1), q(X2))*	Non-linear weakening
Min*(q(X1), q(X2))* < *q(X1 ∩ X2)* < Max*(q(X1), q(X2))*	Univariate weakening
*q(X1 ∩ X2)* > Max*(q(X1),q(X2))*	Bivariate enhancement
*q(X1 ∩ X2)* = *q(X1)* + *q(X2)*	Independent
*q(X1 ∩ X2)* > *q(X1)* + *q(X2)*	Non-linear enhancement

All statistical analyzes were conducted using R (version 3.6.0). A two-sided *p*-value of less than 0.05 was considered statistically significant. The geographic detector method was implemented using R packages and software available on http://www.geodetector.cn.

## Results

### Study participant characteristics

The demographic characteristics of the study participants are summarized in Table 4. A total of 16,171 participants from 122 cities across 28 provinces and autonomous regions in China were included in the study. Of these participants, 49.1% were aged between 45–60 years, and 50.9% were 60 years old or older. Additionally, 46.3% of the participants were male, and 53.7% were female. Geographically, 49.4% of the participants resided in the North, while 50.6% were in the South.

**Table 4:** Characteristics of sample included in the analyses, by age, gender and region

Characteristic	Sample size (%)
Age	
Middle-aged (45 -60)	7,942 (49.1)
Elderly (≥ 60)	8,229 (50.9)
BMI	
< 24	8,725 (54.0)
≥ 24	7,446 (46.0)
Gender	
Male	7,495 (46.3)
Female	8,676 (53.7)
Region	
North	7,928 (49.1)
South	8,243 (50.9)
Urban/rural	
Urban	6,077 (37.6)
Rural	10,094 (62.4)
Education	
Primary school or below	3,424 (21.2)
Middle school	10,806 (66.8)
High school or above	1,941 (12.0)

### The spatial distribution of overweight prevalence rate

**Fig. 1:**
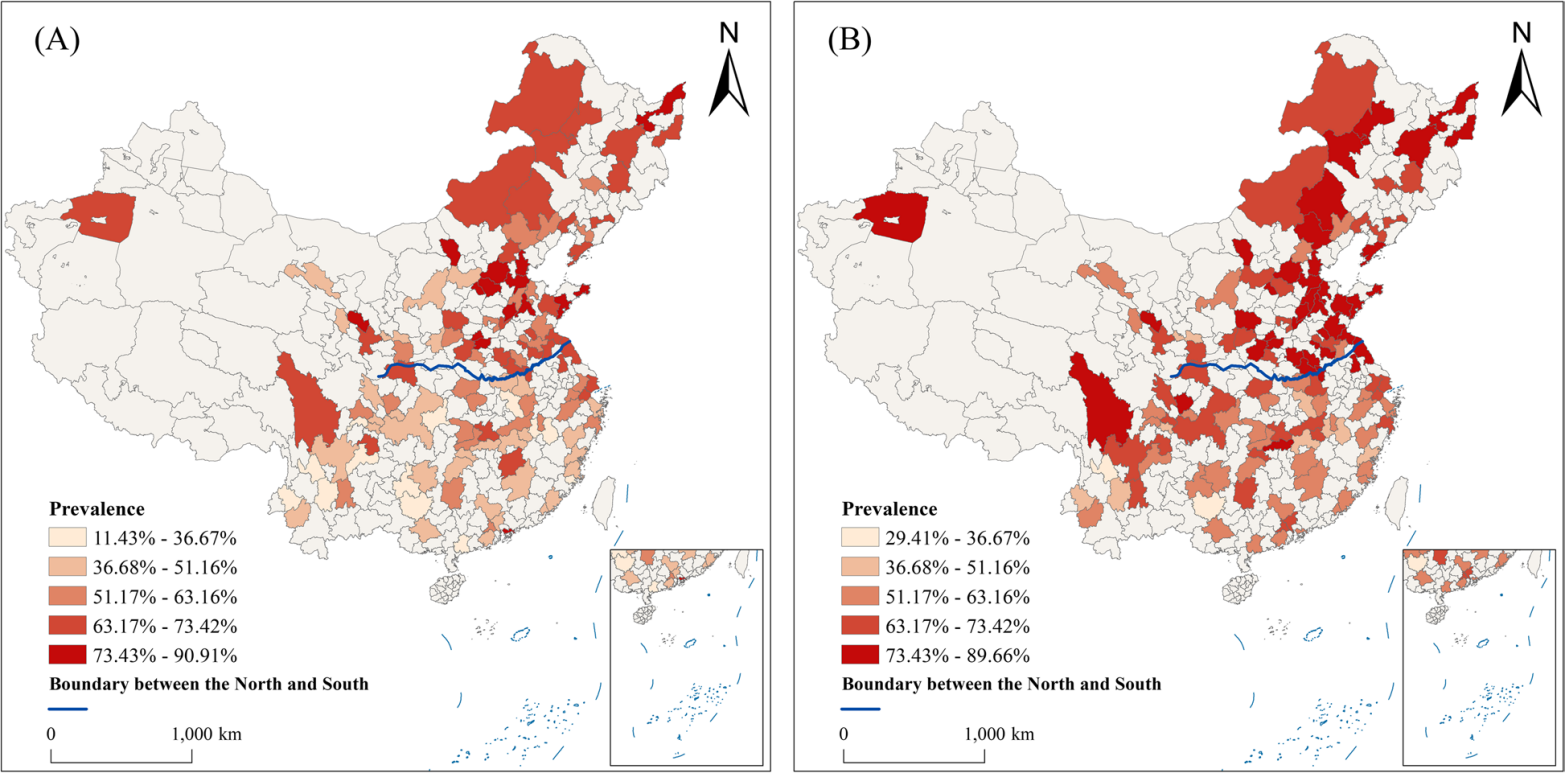
Distribution map of the prevalence of overweight by age (**A**) for middle-aged people, (**B**) for the elderly

**Fig. 2:**
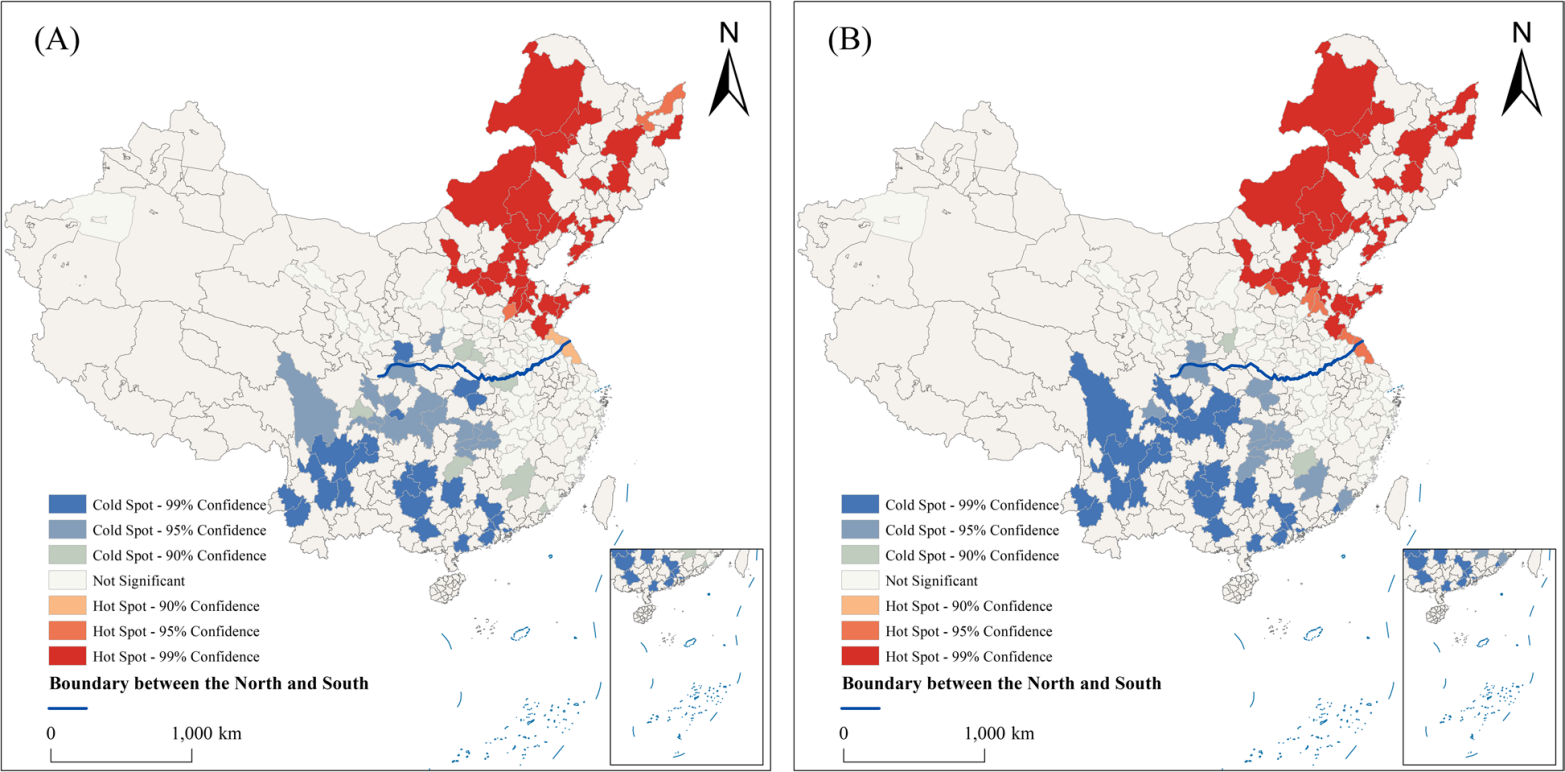
Analysis of cold and hot spots of prevalence of overweight by age (**A**) for middle-aged people, (**B**) for the elderly

The prevalence of overweight is higher among middle-aged individuals than the elderly. In 2015, the percentage of middle-aged and elderly individuals with overweight in each city was 67.27% and 57.39% respectively, as shown in Fig. 1. There is a significant difference in the prevalence of overweight between the north and the south, with rates of 54.86% and 38.75%, respectively. The study revealed clear spatial heterogeneity. The analysis of spatial autocorrelation for overweight rates among middle-aged and elderly individuals in each city shows a significant degree of spatial agglomeration, as presented in Table 5. The Getis-Ord Gi* statistics analysis suggests that the hot spots are primarily in the northeast of China, while the cold spots are located in the southwest, as depicted in Fig. 2.

**Table 5:** Spatial autocorrelation of overweight rate in middle-aged and older Chinese adults

	Elderly	Middle-aged
Moran's I	0.17	0.18
Z-score	14.32	14.83
*P*-value	< 0.01	< 0.01

### Dominant factor analysis

The risk factor analysis demonstrated that the prevalence of overweight is influenced by outdoor air pollution, exhibiting stratified heterogeneity in spatial distribution and age groups. Generally, the q-stasistic of air pollutants associated with overweight prevalence in the southern region were higher than those in the northern region, with the middle-aged group showing higher values compared to the elderly group. Detailed findings of the risk factor analysis for both north and south China, along with the different age groups, are presented in Table 6.

**Table 6:** q-statistic for different air pollutant factors in north and south in China

	PM_2.5_	PM_10_	SO_2_	O_3_	NO_2_
North					
Middle-aged	0.06	0.13	0.31^b^	0.09	0.02
Elderly	0.10	0.12	0.13^a^	0.11	0.11
South					
Middle-aged	0.21^b^	0.23^b^	0.33^b^	0.11^b^	0.16^b^
Elderly	0.17^b^	0.21^b^	0.07^b^	0.07^b^	0.24^b^

^a^ 5% level of statistical significance

^b^ 1% level of statistical significance

In northern China, among the five pollutants SO_2_ had the highest impact on the rate of overweight, with a q-statistic of 0.31 (*p* < 0.01) for the elderly population, while other pollutants influence on overweight are not significant.

The south has a significant relationship between all five pollutants and overweight rates (*p* < 0.01), however, the explanatory power varied across different age groups. In middle-aged people of the south, SO_2_ had the highest explanatory power for overweight rates with a q-statistic of 0.33, while O_3_ had a relatively lower explanatory power with a q-statistic of 0.11. In the elderly population of the south, NO_2_ had the strongest explanatory power with a q-statistic of 0.24, while SO_2_ had a lower explanatory power with a q-statistic of 0.07. Additionally, age-related differences were observed in the effects of fine particulate matter (PM_2.5_ and PM_10_), with higher q-statistic for PM_2.5_ and PM_10_ observed among middle-aged individuals compared to the elderly.

### Interaction effect

**Fig. 3:**
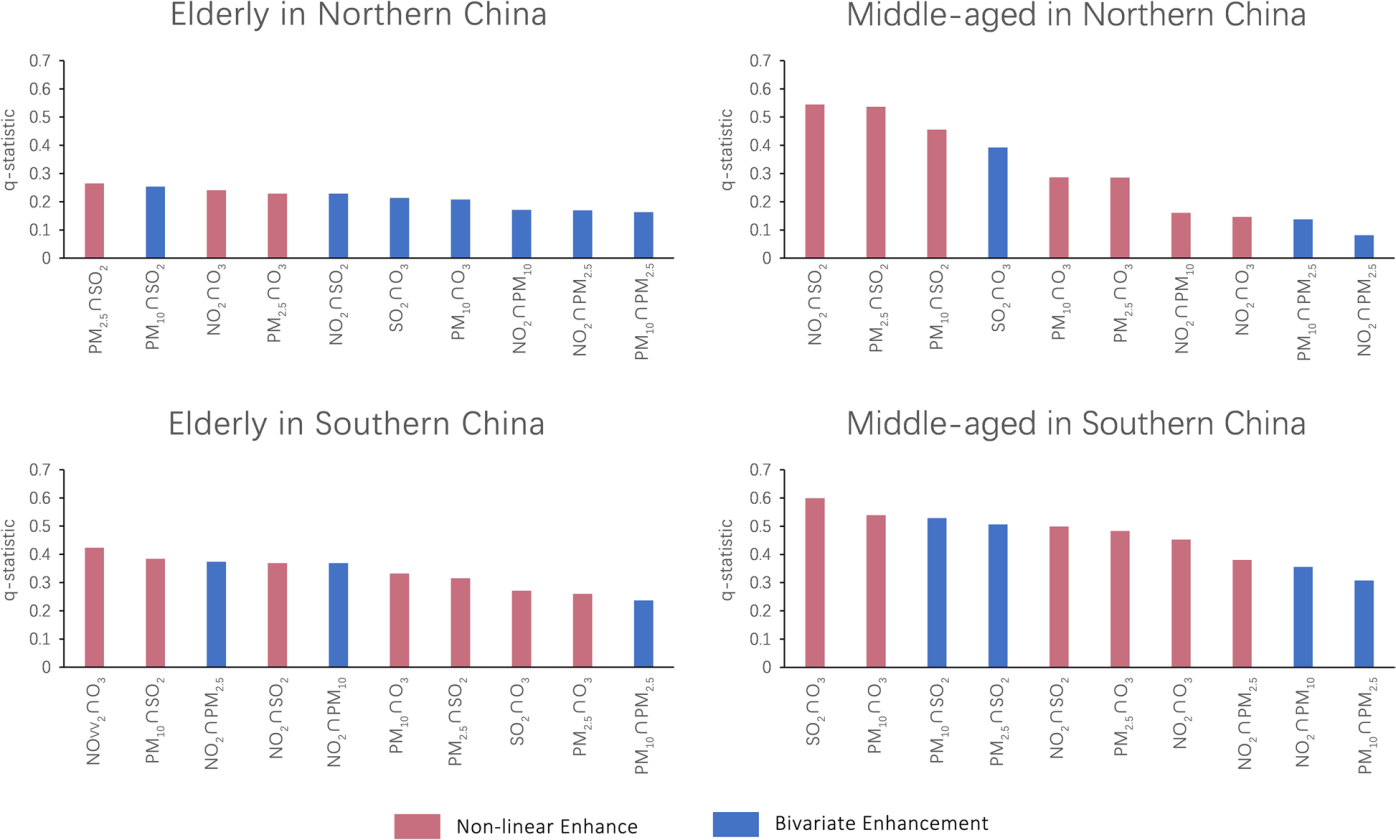
Interaction effect between air pollutants. Note: The x-axis is arranged according to the q-statistic in descending order

Figure 3 illustrates the interaction effects of risk factors on the prevalence of overweight among various age groups in different regions. The explanatory power of all risk factors for the prevalence of overweight had improved after pairwise interaction, overall. Notably, the southern population and middle-aged population exhibit a more noticeable interaction effect.

Among the elderly residing in the northern region, the magnitude of the interaction of various factors demonstrated minimal variation. The interaction with the highest magnitude among this group, with a q-statistic of 0.27, was the interaction between PM_2.5_ and SO_2_, and the lowest was between PM_10_ and PM_2.5_, with a q-statistic of 0.16. The magnitude of the interactions for each factor among middle-aged individuals in the north differed significantly. The largest interaction was between NO_2_ and SO_2_, with a q-statistic of 0.55, while the smallest was between NO_2_ and PM_2.5_, with a q-statistic of 0.08. SO_2_ had the most significant impact and was in the top three positions amid the interactions, including SO_2_ and NO_2_, PM_2.5_, and PM_10_.

Similarly, among the elderly population in the southern region, there was minimal variation in the magnitude of interaction among various factors. The most pronounced interaction among the elderly in the south was between NO_2_ and O_3_, with a q-statistic of 0.42. Moreover, the minimum value of q-statistic for the interaction between PM_10_ and PM_2.5_ was 0.24, which suggests that none of the pollutants had a distinctly notable impact.

While the explanatory power of O_3_ alone was limited for middle-aged individuals in the south, its interaction with other factors was significant. More specifically, the interactions of O_3_, SO_2_, and PM_10_ had q-statistic of 0.60 and 0.54, ranked as the top two, respectively. Moreover, the interaction between PM_10_ and PM_2.5_ had a minimum q-statistic of 0.31.

In summary, although the interactions between different age groups in different regions are different, SO_2_ has played a significant role and is a key pollutant affecting overweight rates. In addition, the influence of O_3_ on the prevalence of overweight alone is small, but the explanatory power is significantly improved by combining with other factors.

## Discussion

Overweight has emerged as a crucial factor that poses a threat to global public health. Although air pollution has been recognized as a risk factor for overweight, its spatial relationship with the same remains ambiguous. We conducted an investigation to assess the impact of outdoor air pollution on overweight among middle-aged and elderly individuals residing in northern and southern China.

The findings revealed significant heterogeneity in the effects of air pollutants on overweight concerning spatial, age, and pollution variables. Firstly, the study observed a more considerable impact of air pollution on the overweight of middle-aged and elderly individuals residing in the southern region, when compared with their counterparts in the northern region. Moreover, the pollutants had differential effects on individuals belonging to different age groups, and the interaction among these pollutants was quite apparent. Furthermore, the results indicated that SO_2_ had a higher impact on middle-aged individuals than the elderly. In contrast, NO_2_ had a greater association with the overweight of aged individuals. Also, the interaction between other pollutants and O_3_ appeared very noticeable.

The study revealed a more significant impact of outdoor air pollutants on middle-aged and elderly individuals residing in the southern region. The warmer and more humid climate in the south when compared with the north intensified the adverse impact of air pollutants on the population through three factors. Primarily, a multitude of studies have demonstrated that higher ambient temperature causes an interaction with air pollutants that aggravates their negative impact on human health. Besides, toxicology experiments on mice have shown that high temperature exacerbates the toxic effects of many environmental toxins [32]. Additionally, the heightened breathing intensity and oxygen demand by the heart during this process result in the inhalation of a greater volume of air pollutants [33]. Further, air pollution has greater limitations on outdoor activities in the southern region. During winter, when air pollution is at its peak [34], the lower temperature in the northern region restricts people's outdoor activities and reduces their exposure time to air pollutants to some extent. Consequently, the annual outdoor activity time in the southern region surpasses that in the northern region, and the former may be more susceptible to the adverse effects of outdoor air pollution.

The study suggests that outdoor air pollution has a more significant impact on overweight in middle-aged individuals than the elderly, despite many studies suggesting that older adults are more vulnerable to the ill effects of air pollution [35–37]. Firstly, this may be due to longer commuting times for middle-aged people, which, according to Zhang, is linked to lower subjective health indicators and higher BMI [38]. Furthermore, Christian notes that extended commuting hours result in changes in individual behaviors leading to weight gain and other health issues [39]. It is essential to be mindful of the impact of air pollution during commuting since Nazelle argues that daily commuting creates a disproportionate increase in urban air pollutant exposure [40]. Air pollution levels are usually highest on commuting routes, particularly during morning rush hour [41]. Exposure to traffic pollution has been found to increase the risk of overweight in specific populations [42]. Secondly, middle-aged individuals' increased work-life pressure might exacerbate the effects of air pollution on overweight. Multiple studies have shown that increasing personal stress raises the risk of being overweight [43, 44]. Dallman suggests that stress reduces the individual's response and cognitive level, stimulating the production of glucocorticoids and insulin, thereby increasing food intake and leading to undesired weight gain [45]. Besides, excessive stress has been associated with poor sleep and changes in serum leptin and ghrelin levels, leading to increased hunger and appetite [46]. Air pollution may thus exacerbate this process as it directly increases the risk of overweight and indirectly affects the individual's psychological health [47].

The study revealed that different pollutants have varying impacts on overweight in different age groups. SO_2_ has a greater effect on middle-aged people, while NO_2_ has a greater effect on the elderly, consistent with previous studies [36, 48, 49]. SO_2_ concentration is more impactful on hypertension in adults below 60 years, and higher SO_2_ exposure leads to increased risk of type 2 diabetes in people aged 30–50 [49, 50]. NO_2_ exposure significantly increases blood lipid levels in the elderly population, and this is related to overweight [51–53]. The study found that the interaction between pollutants significantly increases the explanatory power of overweight. The influence of O_3_ on overweight is initially low but significantly improves after interacting with other pollutants. It indicates that O_3_ has a considerable effect on overweight in middle-aged and elderly people but acts jointly with other pollutants. Although few studies have examined the combined effects of air pollutants on human health, people are typically exposed to multiple air pollutants simultaneously [54]. When considering the interaction of pollutants, the strength and direction of their effect may be opposite. Zhang et al. (2021) showed that particulate matter (PM_2.5_, PM_10_) is stronger when controlling gaseous pollutants (SO_2_, NO_2_, O_3_), and the effects of gaseous pollutants weaken after controlling for particulate matter [55].

Some studies have highlighted the significance of addressing spatial confounding issues when assessing the association between influencing factors and health risks [56, 57]. However, there remains a limited body of research investigating the relationship between overweight and environmental factors. This study utilized a spatial stratified heterogeneity design to mitigate the impact of spatial confounding and to quantitatively analyze the non-linear and interaction effects of influencing factors on overweight, which offers distinct advantages.

The study's limitations should be discussed. Firstly, the individual-scale data was abstracted to the urban scale to study the spatial distribution of overweight and match it with urban air pollution data, which to some extent, concealed individual differences. Secondly, due to the lack of data, we used divisions such as North–South divisions instead of differences in individual diet types, living habits, and socioeconomic status. This approach erased individual differences to a certain extent. Lastly, using BMI to reflect the degree of personal overweight may be controversial since many studies have proposed that Waist-to-hip ratio (WHR) may better reflect the impact of overweight on health [36, 58].

## Conclusion

This study found significant spatial and age differences in the relationship between outdoor air pollution and overweight prevalence in middle-aged and older adults. Air pollution has a greater impact on overweight rates of middle-aged and older individuals in southern China compared with northern China. Moreover, the impact of air pollution varies among age groups, with different pollutants having varying impacts, and some pollutants demonstrating significant interaction effects. SO_2_ has a greater impact on middle-aged individuals, whereas NO_2_ has a greater impact on older individuals. O_3_ has significant interaction effects with other pollutants.

## Data Availability

Original survey datasets from the CHARLS are freely available to all bonafide researchers and can be download from https://charls.charlsdata.com. The air pollution dataset used and analyzed during the current study are available from the corresponding author on reasonable request.

## Abbreviations

BMI: Body mass index
CHARLES: China Health and Retirement Longitudinal Study
CNEMC: Chinese National Environmental Monitoring Center
NO_2_: Nitrogen dioxide
O_3_: Ozone
PM_2.5_: Fine inhalable particles
PM_10_: Inhalable particles
SO_2_: Sulfur dioxide
WHR: Waist-to-hip ratio
